# Cytopathic Mechanisms of HIV-1

**DOI:** 10.1186/1743-422X-4-100

**Published:** 2007-10-18

**Authors:** Joshua M Costin

**Affiliations:** 1 Biotechnology Research Group, Department of Biology, Florida Gulf Coast University, 10501 FGCU Blvd. S., Fort Myers, Fl, 33965, USA

## Abstract

The human immunodeficiency virus type 1 (HIV-1) has been intensely investigated since its discovery in 1983 as the cause of acquired immune deficiency syndrome (AIDS). With relatively few proteins made by the virus, it is able to accomplish many tasks, with each protein serving multiple functions. The Envelope glycoprotein, composed of the two noncovalently linked subunits, SU (surface glycoprotein) and TM (transmembrane glycoprotein) is largely responsible for host cell recognition and entry respectively. While the roles of the N-terminal residues of TM is well established as a fusion pore and anchor for Env into cell membranes, the role of the C-terminus of the protein is not well understood and is fiercely debated. This review gathers information on TM in an attempt to shed some light on the functional regions of this protein.

## Review

### HIV discovery and clinical presentation

In 1981 the CDC (USA) began noting a group of homosexual men presenting with symptoms of a rare opportunistic infections at a San Francisco clinic [1,2]. These patients were later found to be suffering from severe immune deficiency and their syndrome was dubbed acquired immune deficiency syndrome (AIDS). In 1983, two viruses were simultaneously isolated in the United States and France thought to be the cause of these infections, named HTLV-III (Human T Lymphotropic Virus) and LAV (Lymphadenopathy Associated Virus) respectively [3-8]. HTLV-III and LAV, along with a third virus isolated from AIDS patients in San Francisco, named ARV for AIDS-associated Retrovirus [9] were later discovered to be the same virus and renamed Human Immunodeficiency Virus, or HIV [10].

**Figure 1:**
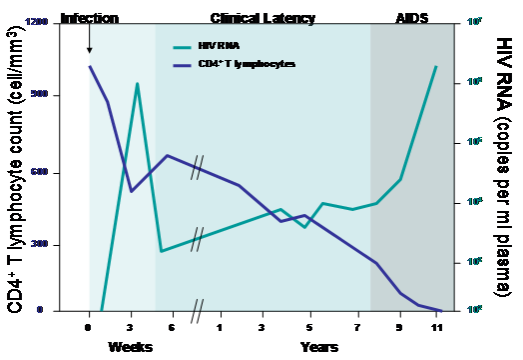
**Time course of HIV infection**. Time course of HIV infection showing correlation of viral load, CD4^+ ^T cell, and CD8^+ ^T cell counts.

Since its discovery it has been estimated that more than 64.9 million people have been infected with HIV world-wide, with greater than 32 million AIDS-related deaths (refer to [222]. Infection with HIV is characterized by three clinical stages – acute viremia, a latency phase of variable duration, and a classification of clinical AIDS (Figure 1). Concurrent with initial infection, virus can be detected in the blood of patients [11,12]. After the initial viremia peaks, the level of virus in the blood falls off and a phase of "latency" ensues. During the latency phase, HIV load is generally very low to non-detectable, though there is a high turnover of CD4^+ ^T cells and HIV virion production [13-17]. Before the advent of highly active antiretroviral therapy (haart), it was established that the levels of virus in the blood at this stage are negatively correlated with prognosis and time course of progression to AIDS [17-19]. It is during the latency phase that CD4^+ ^T cell counts also begin to decline and an inversion of the CD4^+^/CD8^+ ^T cell ratio occurs. A CD4^+ ^T cell count below 200 cells/mm^3 ^and infection with at least one opportunistic infection, such as Pneumocystis Carinii defines clinical AIDS. It is at this final stage where patients' immune systems are no longer able to function properly and patients eventually succumb to their secondary infections, to otherwise rare cancers (such as Kaposi's sarcoma) or to other manifestations of HIV infection (such as neuropathy).

### HIV classification, structure, genome, and replication cycle

**Figure 2:**
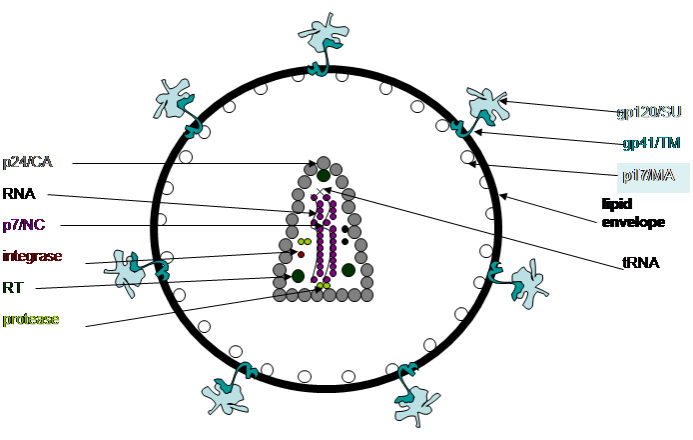
**The HIV-1 virion**. Graphical depiction of the HIV-1 virion. Vpu is not thought to be present in the virion in any appreciable amount.

HIV is enveloped, contains reverse transcriptase and 2 identical copies of a positive sense, linear RNA genome (Figure 2). HIV is classified in a subgroup of retroviruses called the lentiviridae based on these "morphological, genetic, and biological properties" [10,20]. HIV is a slow virus – the clinical "latency" phase can last more than 20 years. During this time, HIV can have widespread effects on immunological and neurological systems. Lentiviruses are known for their cytolytic and immunosuppressive properties and include viruses such as simian immunodeficiency virus (SIV), feline immunodeficiency virus (FIV), caprine arthritis-encephalitis virus (CAEV), and equine infectious anemia virus (EIAV).

**Figure 3:**
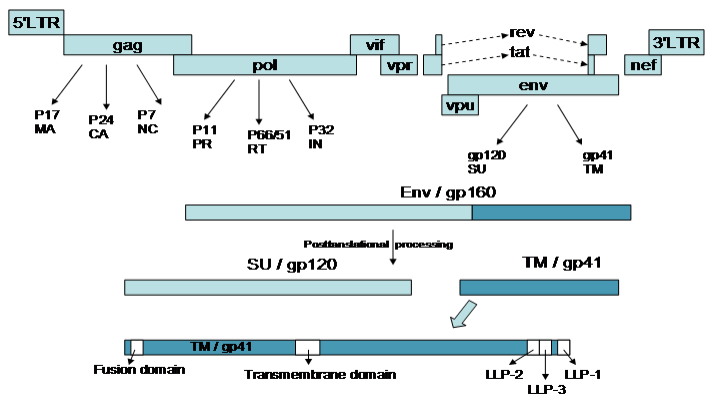
**HIV genome and replication cycle**. Depiction of the ~10 Kb HIV-1 genome showing the organization of genes and their transcriptional splicing (dashed lines). Relevant TM domains are highlighted.

**Figure 4:**
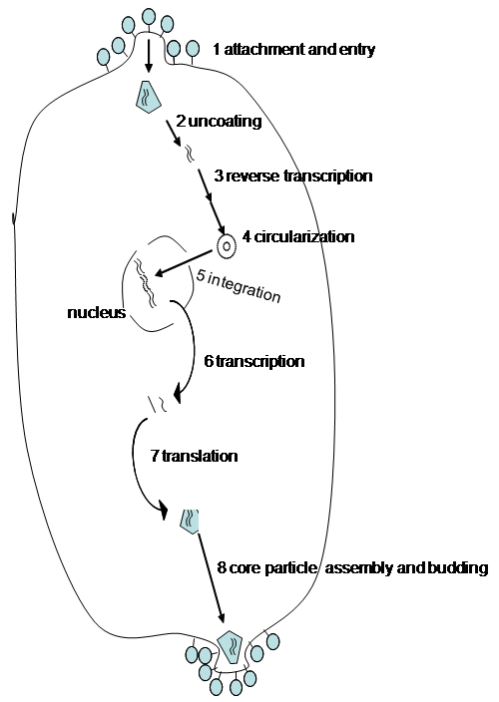
**Overview of the replication cycle of HIV-1**. Overview of some of the basic steps of HIV infection of a cell.

As with all lentiviruses, HIV possesses a complex genome (in this case, 9.8 kb) containing accessory and regulatory genes (Figure 3). An additional, novel open reading frame, *vpu *separates the *pol *and *env *regions [10,21]. In total 9 genes are present that can be classified into 3 functional groups. Gag, Pol, and Env are structural genes; Tat and Rev are regulatory genes; Vpu, Vpr, Vif, and Nef are accessory genes. A general overview of the replication cycle in a single cell is presented in Figure 4. After direct fusion of the virion and cellular lipid membranes, the viral core is released into the cytoplasm where it uncoats and releases the RNA genome. The viral genome is then reverse transcribed and transported to the nucleus where it integrates as a provirus. The early gene products, tat, rev, and nef are first transcribed, followed later by the rest of the HIV genome. Assembly and budding of progeny virions takes place at the plasma membrane.

*Gag *codes for the capsid protein which recruits two copies of the RNA genome, the *pol *gene products (reverse transcriptase, protease, and integrase), and other viral and cellular gene products to the plasma membrane for budding of the virus. *Env *encodes the Envelope protein, or Env, which is synthesized as a single polyprotein in the endoplasmic reticulum. After synthesis, Env (gp160) is heavily glycosylated in the Golgi complex before a cellular protease cleaves it into the noncovalently associated proteins, surface glycoprotein (SU, or gp120) and transmembrane glycoprotein (TM, or gp41).

SU is an extracellular protein which primarily functions to recognize HIV's primary and secondary cellular receptors, CD4 and CCR5/CXCR4 respectively on target cells [22]. Analysis of the expression of these receptors in immune cells is sufficient to explain the tropism of HIV, primarily macrophages and T lymphocytes. TM on the other hand appears to function in membrane interactions. It is an integral membrane protein which contains a transmembrane anchor domain that anchors Env into the lipid membrane [10]. TM is responsible for fusion of the viral and cellular membranes via its fusion peptide located in TM's extracellular, N-terminal domain. The fusion peptide of HIV-1 has shown some structural and functional similarities to the hydrophobic internal region of bovine prion protein (BPrP_tm_) [23]. Both of these peptides are notable for their ability to interact with, and insert into membranes. After the addition of calcium, there is a shift in conformation from α-helix to β-sheet which accompanies membrane fusion. The C-terminal, cytoplasmic tail of TM is known to help direct the assembly of virions at the cell surface [24], among other functions (see below).

The regulatory proteins, Tat and Rev are both RNA binding proteins. Tat is an RNA binding protein and transcriptional activator that works to ensure full length HIV genomes are produced [25]. Tat is also known to activate cellular genes such as TNF-β and TGF-β as well as downregulate the expression of other cellular genes such as bcl-2 and MIP1-α. HIV's other regulatory protein, Rev, is an RNA binding protein that is required for the transition of HIV gene expression from the early phase to the late phase [26]. Rev accomplishes this through binding of unspliced or incompletely spliced viral RNA's in the nucleus and nucleolus and then transporting them into the cytoplasm, leaving fewer viral RNA's to be completely spliced.

The accessory proteins coded in the HIV genome are known to be multifunctional. Nef, or negative factor, has been shown to downregulate existing CD4 and MHC I expression at the cell surface via degradation in lysosomes [27,28]. Nef can perturb T cell activation (up- or down-regulate) and stimulate HIV virion infectivity. Nef shows sequence and structural features of scorpion peptides known to interact with K^+ ^channels. When Nef is added to chick dorsal root ganglion an increase in K^+ ^current is observed [29]. Vpr allows HIV to infect nondividing cells by acting as a nucleocytoplasmic transport factor [30]. Vpr has reported cation-selective ion channel activity in planar lipid bilayers [31]. Vpr "pores" may be active in both nuclear and mitochondrial membranes [32-34]. In the nuclear membrane, Vpr may facilitate the translocation of the HIV-1 preintegration complex from the cytoplasm to the nucleus. In mitochondrial membranes, Vpr binds to the adenine nucleotide translocase (ANT), part of the mitochondrial permeability transition pore (MPTP). Binding of Vpr to ANT can convert it to a pro-apoptotic pore, leading to uncoupling of mitochondrial respiration, loss of transmembrane potential, swelling of the matrix, and release of intermembrane proteins. Additionally, Vpr acts to arrest the cell cycle in the G2 phase, preventing entry into mitosis [35]. The internal membrane localized Vpu functions to downmodulate CD4 expression via ubiquitin-mediated degradation and to enhance virion release through the formation of an ion channel which collapses membrane potential and may promote virion release (discussed in greater depth below) [27]. Finally, Vif is essential for the replication of HIV in PBMC's, lymphocytes, macrophages, and certain cell lines suggesting that it may act through interaction with a cellular factor that is host species specific [26].

### HIV cytopathology and induced ion modifications

Selective depletion of CD4^+ ^T cells is a hallmark of HIV infection and is accomplished, at least in part, due to direct cytopathic effects (CPE) of the virus [36]. The HIV replication cycle is complex and not completely understood. It is increasingly thought to begin via interaction with dendritic cells during transmission [37]. A protein present on dendritic cells, DC-SIGN, reversibly binds HIV, with or without internalizing it, and shuttles it to a regional lymph node, thought to be the primary site for replication and spread of HIV. When the virus encounters a macrophage or T cell with its primary CD4 receptor and a coreceptor, either CXCR4 or CCR5, conformational changes caused by the binding of SU expose the fusion peptide of TM triggering direct fusion of the HIV and host cell membranes. CD4 is expressed on many cells in the body, but is found in highest levels on T lymphocytes, macrophages, and in the brain, primarily astrocytes [38]. The specificity for the coreceptor is determined by the V3 loop region of SU and explains the tropism of the virus for specific cell types [39]. CCR5-utilizing HIV (macrophage tropic, non-syncytium inducing) strains are preferentially transmitted over CXCR4-utilizing (T cell tropic, syncytium inducing) strains for reasons that are not completely understood [40,41]. A naturally occurring ΔCCR5 mutation in humans correlates with resistance to infection by HIV [42]. The emergence of CXCR4 strains during the course of an infection is correlated with increased CD4^+ ^T cell depletion and accelerated progression towards AIDS [43]. This increase in T cell depletion can at least be partially explained by the fact that a higher percentage of T cells express CXCR4 (90–100%) than express CCR5 (10–30%) [44,45] and suggests a role for direct cytopathic effect by HIV.

The ability to directly lyse CD4^+ ^T cells have been postulated to at least partially cause the reduction of these immune effecter cells which leads to the clinical condition of AIDS. Three additional mechanisms have been postulated for CD4^+ ^T cell depletion including immune destruction of infected cells, apoptosis, and impaired lymphocyte regeneration. These alternative mechanisms for *in vivo *CD^+ ^T cell depletion are reviewed in McMichael et al., 2000, Alimonti et al., 2003, and Douek et al., 2003 respectively [46-48]. The relative contribution of each of these mechanisms, if any, is still not clear. However, there is strong evidence that direct cytopathic effects of the virus play a large role in its pathogenicity.

Only cells expressing CD4 along with the proper coreceptor are infected by HIV [38,49]. HIV kills cells in cell culture as well as *in vivo*. Through the course of natural disease, the virus switches use of coreceptors from the less cytopathic CCR5 (R5), non-syncytium inducing (NSI) variants to the more cytopathic CXCR4 (X4), syncytium inducing (SI) variants [41]. The emergence of X4 variants during an infection is associated with an accelerated progression towards AIDS [43]. After the development of Highly Active Anti-Retroviral Therapy (HAART), it became clear that HIV-1 infection was a highly dynamic process involving massive covert replication of HIV-1 in lymphoid tissues at all stages of an infection with continual destruction and regeneration of CD4^+ ^lymphocytes [50]. It is estimated that HIV-infected cells and plasma virions have drastically shortened average life spans in vivo – 2.2 and 0.3 days respectively [14-16,51]. Uninfected T lymphocytes can survive >80 days by comparison [51]. If the estimates of total HIV virion production of 10.3 × 10^9 ^virions a day are correct, then statistically there are enough virions present in an *in vivo *infection to cause massive direct CPE [52,53].

*In vitro*, HIV causes two types of CPE – syncytia and single cell lysis. Syncytia are formed when Env expressed on an infected cell late in infection interacts with CD4 of a neighboring cell, triggering the fusion peptide of TM to fuse the two membranes. Repeated occurrences of this event allows for the formation of giant, multinucleated cells. This type of CPE is thought rarely, if ever to occur *in vivo*, and in fact rarely occurs during infection of human PBL's *in vitro*, with the possible notable exception of the brain [54-56]. HIV patients with AIDS Dementia Complex (ADC) are found to have many giant, multinucleated cells in the brain upon autopsy, mostly consisting of glial cells known to express CD4. In addition to multinucleated syncytial cells, single cells infected with HIV undergo a process termed balloon degeneration whereby cells swell up beyond the limits of their membrane integrity and lyse. This is by far the most common type of CPE observed *in vitro *[10,20,36,57]. Cell swelling in this case appears to be irreversible in most cells, though it has been hypothesized that those cells which can overcome these alterations in cell volume may survive to become a population of chronically infected cells [20]. One factor that both of these types of CPE have in common is increases in cell volume. Though syncytia do not generally lyse, they do show increases in cell volume.

Experimenting with Sendai virus, Micklem and Pasternak, 1977 observed that alterations in the plasma membrane of infected cells occurred within minutes of adsorption of the virus [58]. These alterations included: changes in intracellular ion concentrations, osmotically driven water entry, and an increase in cell volume [59,60]. Basford et al., 1984 hypothesized that after direct fusion of Sendai virus lipid membrane with the host cell, the viral lipids and proteins introduced into the host cell were able to perturb the membrane in a manner reminiscent of the bee venom melittin [61]. In the case of HIV, Grewe et al., 1990 noted that early interactions of HIV with host cell membranes were similar to those observed with Sendai virus [62]. Further evidence provided by Rasheed et al., 1986 showed that HIV was able to cause CPE as an early event. UV-irradiated HIV, lacking the ability to replicate but still able to infect cells by direct fusion of its lipid membrane to the host cell still caused single cell balloon degeneration of the RH9 T lymphoblastoid cell line. Cloyd and Lynn, 1991 further proved that the permeability of the host plasma membrane was enhanced early (12–24 hours) post infection to small molecules such as Ca^2+ ^and sucrose, with greater permeability seen later (24–72 hours) post infection [54].

Viral ion channels, or viroporins, are present in many lytic animal viruses. The cellular plasma membrane maintains cellular materials and ionic gradients necessary for the proper functioning of the cell. The ability to alter intracellular ion concentrations is necessary for many of these animal viruses in their life cycles and is a common theme of cytolytic viruses [63,64].

HIV infection causes increases in intracellular monovalent cations during infection analogous to what has been observed for other animal cytolytic viruses, such as poliovirus and sindbis virus. Acute infection of RH9 cells, a T-lymphoblastoid cell line, with HIV-1_HXB2_, a lab adapted strain, increases intracellular Na^+ ^and K^+ ^concentrations as measured by ion sensitive dyes [65,66]. The flow of the osmotically active monovalent cations, K^+ ^and Na^+ ^into infected cells correlates with CPE. Increased intracellular ion content is expected to be associated with increased water influx into the cell to balance osmolarity, thereby expanding the total volume of both single and syncytial cells. Furthermore, strains of HIV known to be more cytopathic, the syncytium inducing (SI) strains, induced greater increases in [Na^+^]_i _and [K^+^]_i _than did non-syncytium inducing (NSI) strains of HIV [66]. This correlation remained when primary isolates of HIV were used in place of the lab adapted strain, and when primary human PBMC's were used in place of immortalized RH9 cells [66].

Addition of loop diuretics such as bumetanide and furosamide, specific inhibitors of the Na^+^/K^+^/2Cl^- ^cotransporter, at least partially blocked increases in [Na^+^]_i _and [K^+^]_i _levels, suggesting that HIV alters this transporter's normal function in cell volume control [67]. Makutonina et al., 1996 observed a concomitant decrease in pH, from pH 7.2 in uninfected cells, to pH 6.7 in HIV-infected RH9 cells using a pH sensitive dye [68]. Use of the Na^+^/H^+ ^antiporter inhibitor amiloride did not further decrease HIV infected cell pH_i_, but did decrease control cells. This implies that HIV may be inhibiting the Na^+^/H^+ ^antiporter in some manner. The authors further suggest that the increases in [Na^+^]_i _observed during infection may itself lead to this shutoff as it would be unfavorable to exchange an extracellular Na^+ ^for an intracellular H^+ ^when the [Na^+^]_i _is already high.

Some viruses alter intracellular ion concentrations in order to get their mRNA's preferentially translated. Cellular mRNA's are only functional within a narrow range of intracellular ion concentrations, while viral RNA's have been shown to be more resistant [69-73]. Previous studies involving animal cytolytic viruses have shown that altering the external ion concentration can affect internal ion concentrations and pathogenesis of the virus. Altering the external concentration of K^+ ^in the medium of HIV-infected RH9 cells alters the cytopathicity of HIV [65]. Decreasing [K^+^]_e _to zero abrogates visible CPE in cell culture and lowers HIV protein translation by 40–50%. Alternatively, increasing [K^+^]_e _from 5 mM (normal) up to as much as 75 mM increases visible CPE and increases HIV protein translation as much as three fold. Altering [K^+^]_e _with primary human PBMC's has an even greater effect on CPE and protein translation than it had with cell culture. Alteration of the external Na^+ ^concentration did not affect CPE or HIV protein translation [65]. For comparison, increased [K^+^]_e _does not increase poliovirus or Sindbis virus production or CPE [69,70].

### Selected Literature review of viral membrane permeability altering proteins

Increased membrane permeability caused by viroporins, glycoproteins, and proteases is a typical feature of animal virus infections [63]. Viroporins are virally encoded, small (generally ≤120 amino acid residues) membrane proteins that form selective channels in lipid membranes. These channels are less discriminating than the highly selective ion channels of bacteria and eukarya and have been hypothesized to be a family of primordial proteins which predate the latter [27]. Features common to viroporins include: promoting the release of virus, altering cellular vesicular and glycoprotein trafficking, and increasing membrane permeability. Amphipathic α-helical domains of viroporins generally oligomerize to form the channel by inserting into lipid membranes with the hydrophobic residues oriented towards the lipid bilayer and the hydrophilic residues facing in towards the lumen of the channel. Though viroporins are not essential for virus replication, they may be necessary for full pathogenesis *in vivo *as they are known to enhance virion production and release [64,74,75]. Many lytic viruses employ altered [ion]_i _(intracellular ion concentrations) in various stages of their replication cycles. This can include steps such as uncoating, host cell translation shutoff, and release of virions from infected cells. Viroporins are not the only strategy viruses employ to alter [ion]_i _– other strategies include generalized membrane destabilization and alteration of existing ion channel and pump functions or expression [20,63,64].

#### Influenza virus

The prototype viroporin, M2 protein, was first isolated from the influenza A virus. M2 protein is one of three proteins found in the virion envelope and is present in less abundance than either of the other two envelope proteins, hemagglutinin (HA) and neuraminidase (N) [76]. Early studies to block influenza A virus infection showed that the virus was sensitive to the compound amantadine at two stages of its replication cycle [77,78]. The first block occurs early in infection after attachment, but before uncoating. As a consequence of this block, a buildup of nondissociated matrix (M1) and ribonucleoprotein (RNP) occurs in endosomal compartments [79,80]. The second block occurs late in infection and inhibits the release of virions [81]. At this late stage of infection, amandatine causes a buildup of HA protein during transport through the *trans *Golgi network that has undergone the acid-induced conformational changes normally observed with viral entry.

Sequencing of viruses with amantadine resistance mapped the mutations responsible for resistance to the transmembrane domain of the M2 protein, a highly conserved protein, even across human, swine, equine, and avian strains of influenza A virus [78,82]. The transmembrane domain of the M2 protein models to form amphipathic α-helices that associate minimally as homotetramers in membranes, forming an ion channel [81,83]. Expression of M2 RNA in *Xenopus *oocytes and analysis of whole cell currents showed a channel selective for monovalent cations that was activated by low pH [84], though later experiments showed the channel to be ~1.5 – 2.0 × 10^6 ^more selective for H^+ ^than Na^+ ^[85]. Mutations in the membrane spanning domain of M2 that conferred amantadine resistance also decreased the conductance of these variant M2 proteins when expressed in *Xenopus *oocytes. Purified M2 protein, as well as peptides corresponding to the TM region of M2, produced an increased conductance of planar lipid bilayers at low pH that was able to be blocked by the addition of amantadine [86,87]. It was then theorized that the M2 protein acts after receptor mediated endocytosis to acidify the interior of the virion and dissociate the matrix protein from the ribonucleoprotein (the first block seen with amantadine), allowing the ribonucleoprotein (RNP) to enter the cytoplasm. The M2 protein was also theorized to work late in infection to prevent Golgi vesicle acidification. This prohibits a premature change in conformation of the HA protein (the second block seen with amantadine), which would halt the assembly of virions. It is important to note that viruses deficient in M2, while severely delayed in growth kinetics are able to undergo multiple rounds of replication in cultured cells. Thus the M2 protein is not essential for influenza A virus replication, but does enhance viral productivity [64,82,88].

A protein analogous to the M2 protein of influenza A virus was discovered in the influenza B virus genome. The NB protein (a.k.a. – BM2) shares many characteristics with the M2 protein. Peptides corresponding to the predicted transmembrane region form α-helices and increase the conductance of lipid bilayers [89,90]. This conductance is inhibited by amantadine, though at a higher concentration than is necessary for the M2 protein of influenza A virus [91]. Purified whole NB protein also increases the conductance of lipid bilayers in a fashion similar to the TM region peptides [92]. NB protein expressed in either *Xenopus *oocytes or mammalian cells form a proton selective channel that is presumably used in a manner analogous to the M2 protein of influenza A virus; for acidification of the virion during uncoating in the endosomal compartment and to equilibrate Golgi vesicles to prohibit premature acid-induced conformational changes in the HA protein of influenza B virus [93]. Single amino acid mutations in the transmembrane region of the NB protein abrogate proton selectivity of the channel, further supporting an analogous role for NB in influenza B virus infections [94].

Early evidence suggests that influenza C virus also encodes an ion channel (CM2) that is a minor virion component [95]. CM2 protein has been shown to possess an α-helical transmembrane domain similar to both the M2 and NB proteins discussed above [96]. However, expression of CM2 protein in *Xenopus *oocytes shows a voltage-activated, Cl^-^-selective ion channel that was not activated by low pH, nor was it inhibited by even high (1 mM) concentrations of amantadine [97]. Studies involving influenza C virus uncoating do not show a dependence on low pH to dissociate the matrix and ribonucleoproteins. At the present time it remains unclear how CM2 protein functions during influenza C virus infection.

#### HIV

Viral protein U (Vpu) of HIV-1 (and SIV_cpz_) is an integral membrane protein found predominantly in the endoplasmic reticulum (ER) and Golgi. It is possibly found to a lesser extent the plasma membrane, but does not seem to be present in the virion [98,99]. Vpu is expressed late in infection as a bicistronic RNA that also codes for the Env protein, which is differentially spliced to produce each protein (see Figure 3). HIV-1 virions deficient in Vpu are impaired in their ability for correct assembly and release. A large proportion of these mutant virus particles displaying altered size and shape from wild type virions remain attached to the cell surface [75]. Vpu possesses two functional domains known to enhance the release of virions from infected cells. The C-terminal cytoplasmic tail of Vpu functions to enhance the degradation of CD4 in the ER [100]. Vpu does not accomplish this task directly, but instead binds CD4 and β-transducin repeats-containing protein (β-TrCP), forming a ternary complex. Formation of this complex requires two phosphorylated serine residues (52 and 56) of the Vpu cytoplasmic tail and targets CD4 for proteolysis using the ubiquitin-dependent proteosome pathway [27,101,102]. It is thought that decreasing the level of expression of CD4 decreases the formation of CD4:Env complexes in the ER, allowing for increased levels of Env expression on the plasma membrane. Increased levels of Env expression at the plasma membrane in turn increases the frequency of virion budding.

The N-terminus of Vpu contains a string of hydrophobic amino acid residues that are predicted to form an α-helical secondary structure and span the ER membrane [90]. This predicted structure is supported by experimental evidence employing solution and solid-state NMR spectroscopy, as well as CD spectroscopy [102-104]. The presence of a functional transmembrane domain of Vpu is correlated with an enhanced rate of release of virus [105].

When Vpu is expressed in *E*. coli, *Xenopus *oocytes, or incorporated into lipid bilayers an increased conductance across each of these membranes is observed [102,105,106]. Analyses of conductances observed in the presence of altered extracellular cation concentrations in these studies suggest that Vpu is selective for monovalent cations. Expression of Vpu with a scrambled transmembrane sequence ablated the increased membrane conductance of lipid bilayers and *Xenopus *oocytes [105]. Just how altering the intracellular ion concentration ([ion]_i_) of the ER and/or Golgi enhances the release of virus particles is still unclear. It has been hypothesized that a collapse of the membrane potential at various points (ER, mitochondrial, and/or plasma membranes) could help to promote virion fusion and release [27], though how this works mechanistically has yet to be worked out.

Incorporation of only the transmembrane domain of Vpu was sufficient to increase planar lipid membrane conductance, whereas expression of the C-terminal intracellular domain did not [102,105]. However, addition of the two amphipathic α-helices just C-terminal to the transmembrane domain, and surrounding the two serine residues necessary for the CD4 degradation function of Vpu seems to promote the oligomerization of Vpu in membranes as well as stabilize the conductive state of the channel [102]. Vpu oligomerizes minimally as a four-helix bundle, but most likely as a five helix bundle [107,108]. Tryptophan residues at position 22 are thought to situate their headgroups into the lumen of the channel, creating a narrow constriction or gate in the closed form of the channel. Rotation of the hydrophobic tryptophan residues around the helical axis is thought to create a more open structure and expose polar serine residues at position 23 in the open state of the channel, allowing monovalent cations to selectively flow through the channel.

Alternatively, Vpu could be interacting with an endogenous ion channel to alter its normal function and modify membrane conductance. Coady et al., 1998 report that expression of Vpu in *Xenopus *oocytes decreases membrane conductance by decreasing expression of an unidentified endogenous membrane channel via degradation in the ER [109]. Furthermore, these authors purport that the increased membrane conductance observed in previous studies was an artifact of the injection of large amounts of RNA and that randomization of the TM sequence also served to ablate its ability to interact with the endogenous ion channel. Expression of exogenous proteins in *Xenopus *oocytes has been shown to sometimes induce non-specific conductances [110]. In support of this theory, Hsu et al., 2004 show that Vpu can physically interact with and inhibit TASK-1, an endogenous mammalian K^+ ^channel [111]. Though the results using planar lipid bilayers in the absence of all proteins except Vpu argues against the conclusion that Vpu conductance is solely caused by interaction with endogenous channels, it does not eliminate this possibility as Vpu's primary mode of action or that Vpu may employ both modes of action.

#### Sindbis virus

Sindbis virus, a member of the family Togaviridae is an enveloped and positive sense RNA cytolytic virus of animals. Sindbis virus is known to increase and decrease the intracellular concentration of Na^+ ^and K^+ ^respectively [112,113]. Late in Sindbis virus infection there is a massive shut-off of host protein translation. An increase in [Na^+^]_i _correlates with the shutoff of host protein synthesis, though Sindbis virus protein synthesis continues and appears to favor these intracellular ionic conditions to force its proteins to be preferentially expressed over host cell proteins.

The cause of the observed increase in membrane permeability appears to be an accessory protein named 6K protein. 6K protein has many similarities to Vpu of HIV-1 – they are small (~60 amino acid residues) hydrophobic, α-helical proteins that associate with membranes [114]. Viruses deficient in 6K protein are replication competent, but are deficient in virion budding [74,115]. 6K protein is produced in the ER and is post-translationally cleaved from the virion glycoproteins E1 and E2. All three proteins are then transported via the Golgi to the cell surface, but 6K protein is not incorporated into virions. 6K-deficient sindbis virus mutants are at least partly restored by the expression of Vpu in trans [116].

6K protein increases membrane permeability to the translation inhibitor Hygromycin B in eukaryotic cells [115]. Inducibly expressed in E. coli, 6K protein induces leakage in the bacterial cell membrane and cell death [117]. Incorporated into planar lipid bilayers, 6K proteins (produced in E. coli or synthetically derived) increase membrane conductance and form cation selective ion channels that are reversibly inhibited by polyclonal antibodies [118].

Wengler et al., 2003 reported the identification of another possible pore that Sindbis virus uses during uncoating that resides in the virion [119]. Sindbis virus enters the cell by way of binding to a cellular receptor to induce uptake into endosomal compartments [63]. Upon acidification of these compartments, the E1 glycoprotein undergoes conformational changes that allow for the formation of a proposed "fusion pore". This pore is of sufficient size to allow the capsid to enter the cytoplasm to begin uncoating, a process that is facilitated in Sindbis virus by a more acidic pH during interaction of the core with the 60S ribosome [120,121]. Therefore it is proposed that the already formed fusion pore also allows H^+ ^ions to exit the endosome, creating a localized area of lower pH that facilitates disassembly of the core while not creating globally acidic conditions in the cytoplasm that would destabilize capsids assembled late in the viral life cycle for budding of progeny virus [119].

In support of this idea, Nieva et al., 2004 recently reported the identification of a membrane permeabilizing region of E1 protein of Simliki Forest virus (a related alphavirus) capable of permeabilizing E. coli as efficiently as 6K protein [122]. The authors suggest that this E1 domain may additionally act as a backup membrane permeabilizing protein to allow budding at the cell surface.

#### Hepatitis C virus

Hepatitis C virus (HCV), a hepacivirus of the family Flaviviridae, encodes a 63 amino acid non-structural protein, P7, that is required for the formation of infectious particles and resembles the 6K protein of sindbis virus [123,124]. When peptides corresponding to the P7 protein are mixed with planar lipid bilayers, ion channels of variable conductance were detected [125,126]. These channels were discovered to be selective for Ca^2+ ^over Na^+ ^and K^+^. Amantadine, a known inhibitor of the influenza virus M2 ion channel, as well as hexamethylene amiloride, a known inhibitor of the HIV-encoded Vpu, both inhibited P7 in planar lipid bilayers [125,126]. In fact, amantadine has shown some efficacy in clinical trials when given in conjunction with the current treatment regimen of IFN-α and ribavirin.

Sequence analysis shows that P7 contains two domains separated by a hydrophilic stretch of amino acid residues which are expected to span the membrane as an α-helix in an "α-loop-α" motif [123]. Expression of P7 in HepG2 cells followed by crosslinking and analysis via Western blot shows the formation of hexameric complexes. In good agreement, transmission electron microscopy of negatively stained E. coli expressing P7 shows ring structures with a diameter consistent with a hexameric arrangement of proteins [126].

Though it has been observed as being present in small amounts in the plasma membrane, P7 protein is mostly localized to the ER, where it presumably would function to release intracellular calcium stores. P7 from bovine viral diarrheal virus (BVDV; a related pestivirus) is known to facilitate virion release from the plasma membrane. BVDV lacking P7 still replicates, but does not produce infectious virions [124]. When P7 protein is added back *in trans*, infectious virions are detected. It has been suggested that P7 from HCV may serve a similar function, though the mechanism of the release of calcium from intracellular stores is as of yet unclear. These studies are complicated to perform directly in HCV due to the inherent difficulty of culturing HCV *in vitro*.

#### Poliovirus

Poliovirus, a non-enveloped virus and a member of the family of Picornaviridae alters intracellular monovalent ion concentrations during infection. An increased total cell volume correlating with increased [Na^+^]_i _and decreased [K^+^]_i _is detected after a couple hours post infection with poliovirus, and has the effect of decreasing the overall rate of protein synthesis of infected cells [112,113,120]. Mammalian cells are known to be sensitive to changes in intracellular monovalent ion concentrations during translation of cellular mRNA [69,127]. Certain viral mRNA, including poliovirus mRNA, has been shown to be less sensitive to altered intracellular cation concentrations. This presents a mechanism by which viruses coax the host cell to preferentially translate viral RNA over most cellular RNA. A decrease in the [NaCl] or an increase in the [KCl] in the medium of infected cells is able to compensate for the induced alterations of intracellular monovalent cation concentrations and allow infected cells to resume normal protein synthesis [128].

The first evidence for a particular poliovirus protein responsible for altering intracellular ion concentrations came from the study of a replication competent poliovirus possessing a mutation in its 2A protease [128]. Expression of individual poliovirus proteins using vaccinia virus in HeLa cells identified the 2B protein (just downstream of the 2A protein) as being responsible for actually increasing membrane permeability [129]. Expression of 2B and 2BC (a precursor protein that stably exists in poliovirus infected cells, some of which is cleaved to produce 2B and 2C), but not any other poliovirus proteins increased HeLa cell permeability to hygromycin B. Mutations in the 2C region of 2BC did not seem to affect its ability to increase plasma membrane permeability suggesting that the 2B region is primarily responsible for this task.

Analysis of the overall hydrophobic 100 amino acid residues present in the sequence of 2B reveals that it contains two predicted α-helical regions separated by a stretch of hydrophilic amino acids [130]. Most of the N-terminal α-helix is amphipathic, while the C-terminal α-helix contains hydrophobic residues are expected to form a transmembrane domain. 2B induces leakage of large unilamellar vesicles (LUV's) composed of phosphatidylinositol in an ANTS/DPX assay [130]. Mutation of various positively charged amino acids in the amphipathic α-helix domain known to decrease membrane permeability to hygromycin B during infection decrease the amount of observed leakage in ANTS/DPX assays. 2B pores allow free diffusion of compounds up to approximately 1000 Da into or out of LUV's. Fluorescence resonance energy transfer (FRET) microscopy shows multimerization in the presence of phosphatidylinositol. Western blot analysis showed these multimers to be SDS-resistant tetramers. Yeast 2-hybrid assays, GST pulldown assays, and FRET microscopy in single living cells have all been in agreement that 2B oligomerizes to form a pore [131-133]. Nieva et al., 2003 modeled the 2B protein to oligomerize in such as way as to form a "barrel-stave"-like pore, where the four amphipathic domains have the hydrophilic residues facing the lumen of the pore and the transmembrane domains surrounding these domains to form a transmembrane anchor.

There have been conflicting reports on the intracellular location of 2B – it has been reported to reside in the ER and Golgi, as well as the plasma membrane [64,134-138]. Concurrent with increased monovalent ion concentration during poliovirus infection, there is a profound rearrangement of the ER and Golgi to the point where the Golgi becomes unrecognizable and numerous membrane vesicles fill most of the cytoplasm late in infection. Whether the 2B protein resides in the plasma membrane to indirectly affect the ER and Golgi, or resides in the ER and Golgi having an indirect affect on the plasma membrane, or is present in all three to produce its effects is unclear. More research needs to be done in this area to distinguish between these three possibilities.

It has been speculated that the capsid of poliovirus is able to form a pore through which the virus is able to enter cells for infection. 160S particles (intact infectious poliovirus) possess a capsid comprised of four proteins – VP1–VP4. VP1–VP3 make up the outer shell of the icosohedrally shaped capsid with their N-termini situated on the inner surface where the entire VP4 protein resides [139,140]. After poliovirus interacts with the Poliovirus Receptor (PVR), there is a rearrangement of capsid proteins such that the N-terminus of VP1 relocates to the outer surface and VP4 is lost from the virion, creating 135S particles. The N-terminus of VP1, which models to form amphipathic α-helices, and VP4, which localizes to the cellular membrane after attachment primarily through a myristolated amino acid residue, are then thought to form a pore or ion channel. Increased conductances across model lipid membranes after addition of 135S particles were measured by Tosteson et al., 1997 and proved to be consistent with this hypothesis [140-142].

#### Rotavirus

Rotavirus (RV) encodes two suspected ion pores that act in different stages of its replication cycle. RV infects the gastrointestinal tract and is a significant cause of diarrheal disease in infants, but does not cause diarrhea in infected adults. A single protein, nonstructural protein 4 (NSP4) has been identified as causing diarrhea and was the first virally encoded enterotoxin identified [143-145]. A peptide corresponding to NSP4 amino acid residues 114–135 is also capable of evoking diarrhea in mice, albeit to a lesser extent than the full protein. Circular dichroism shows this peptide to form α-helices and partition into model lipid membranes and is thought to be the lipid binding domain of NSP4 [146]. Addition of NSP4 protein to gastrointestinal epithelial cells evokes intracellular calcium mobilization most likely from the endoplasmic reticulum (ER). This in turn triggers halide movement across the plasma membrane in what is thought to be the age-dependent step. Finally, transepithelial movement of Cl^-^, followed by Na^+ ^and water into the lumen occur [145]. This secretory diarrhea occurs independent of cyclic nucleotides and the CFTR and in the absence of inflammation. The crystal structure for NSP4 has been solved and predicts that NSP4 could form a homotetrameric pore to potentially span the ER and act as a calcium channel [147]. While this theory has yet to be directly tested, there are a couple facts which suggest this possibility: 1) the predicted hydrophobic interior of the NSP4 pore contains a calcium-binding domain and 2) NSP4 does not alter plasma membrane calcium permeability, but does alter calcium release when expressed within cells.

Rotavirus is nonenveloped and is thought to enter cells through direct penetration. VP4, a structural protein present on the surface of the virion, is cleaved into VP5 and VP8 after treatment with trypsin or after uptake into early endocytic vesicles. Golantsova et al., 2004 report that VP5 has two discrete domains used to penetrate into the cytoplasm. The first domain directs peripheral membrane association, while the second permeabilizes, but does not lyse membranes [148]. VP5 is thought to form transient and size-selective lipidic pores ("ion flicker pores") which allow small molecules to pass. The presence of this pore in an early endocytic vesicle containing a rotavirus virion could allow the [Ca^2+^] in the vesicle to drop – the first step needed for uncoating of the virion and eventual penetration of the virion into the cytoplasm of the cell.

### Existing evidence that the LLP domains of TM HIV may constitute a viroporin

Though it is thought that HIV contains at least one viroporin in Vpu, there is evidence that it codes for more than one. First, Vpu is not present in virions, but membrane perturbations leading to increased intracellular ion concentrations may be an early event in HIV infection [36,54]. Addition of UV-inactivated virus to RH9 T-lymphoblastoid cells, which can attach to and enter cells, but cannot replicate, causes syncytium formation and single cell balloon degeneration. These cytopathic effects – syncytial cell formation, balloon degeneration and cell death – are all observed in the absence of reverse transcription and provirus formation [36].

Ultrastructural analysis of RH9 cells infected with intact HIV virions illustrates partial separation of lipid bilayers, formation of distinct "pores", perturbations, or membrane thickenings within one hour of exposure [149]. Concurrent with these plasma membrane observations were observations of extensive cytoplasmic vacuolization of the endoplasmic reticulum (ER). Vacuolization was most prominent in cells with the highest numbers of bound virions. The authors hypothesize that the cell may be pumping excess ions into the ER, which is followed by water to osmotically balance the ER lumen. This could direct the maintenance of total cell volume in the early stages of infection after disruption of the plasma membrane and resulting ion influxes. Both of these studies indicate the involvement of an actual virion component in CPE.

Analysis of the Env protein, the major protein present in the virion envelope, led to the discovery of 2 domains in the extreme C-terminus of the long (~150 amino acid) cytoplasmic tail of TM that have a high hydrophobic moment [150]. These domains were identified on the basis of their structural motifs and similarities to several natural cytolytic peptides, such as magainin-2 and were given the names LLP-1 and LLP-2 [150,151]. A third domain located between the first two, LLP-3, was discovered later [152]. Magainins are hemolytic, but at concentrations 1–3 orders of magnitude higher than is needed for bactericidal activity [153]. Analysis using the patch clamp technique identified magainin-2 as a voltage-dependent ion channel [154]. Biochemical analyses yielded insights into the mechanism of action of magainin-2. This peptide is cationic, amphipathic, and adopts an α-helical secondary structure in the presence of lipid [153,155]. Molecular modeling studies supported by experimental evidence suggested that the activity of magainin-2 is tied to its ability to form a multimeric structure after insertion into lipid membranes [156,157]. Similar structure-function relationships have been discovered for other natural lytic peptides, such as the cecropins of the North American silk moth, *Hyalophora cecropia*, and melittin from the venom of the honey bee, *Apis mellifera *[157,158].

**Figure 5:**
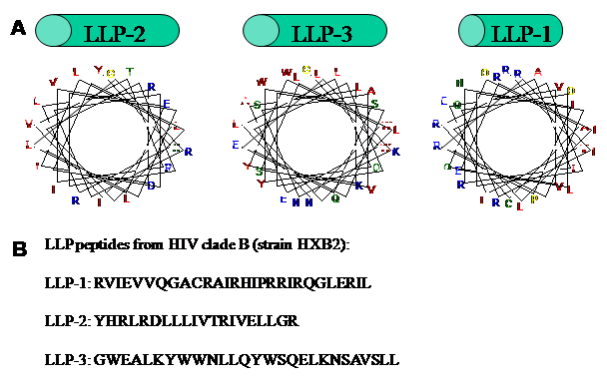
**The LLP domains**. (A) Helical wheel diagrams showing the amphipathic nature of each LLP domain. The coloring scheme is from Benner et al. and graphs were generated using a java applet available at the PredictProtein server [221]. (B) Primary amino acid sequence of LLP peptides which correspond to the LLP-1, -2, and -3 domains of the TM protein are given from the helical wheels in (A).

Figure 5A contains helical wheel diagrams of each LLP domain from the HXB2 strain of HIV-1, as well as their primary amino acid sequence (Figure 5B). When plotted as α-helices, it is apparent that all three domains are amphipathic, generally with hydrophilic residues (colored blue) clustered on one face of the α-helix and hydrophobic residues (colored red) clustered on the opposite face. LLP-3 differs from LLP-1 and -2 in that it lacks the positively charged residues on its hydrophilic face. This secondary structure is conserved across HIV-1 clades, though primary amino acid identity is not [151].

As discussed in Figure 5, helical wheel diagrams suggest these regions have a high propensity to form amphipathic α-helices [150]. Not only are the structure of these LLP-1 and -2 domains highly conserved across all strains of HIV-1, they are conserved across other lytic lentiviruses such as HIV-2, SIV, and equine infectious anemia virus (EIAV) as well, though primary amino acid sequence does vary and as such is not conserved [151]. Closely related nonlytic oncoviruses, including murine leukemia virus lack these conserved amphipathic α-helical structural motifs, though they contain similarly long cytoplasmic tails. The structure of LLP-1 and -2 domains resemble proteins of ion-selective channels, such as the S4 domain of K^+ ^channels, as do the natural cytolytic peptides of the honey bee (melittin) and amphibians (magainins) [150,151,158,159]. Based on analogy to other lytic peptides with similar secondary structure such as magainin-2, and on the observation that when in an anti-parallel arrangement, LLP-1 and -2 exhibit charge complementarity, it has been hypothesized that LLP-1 and LLP-2 could aggregate in lipid membranes with their charged (mostly arginine) residues facing towards each other and their hydrophobic residues facing out toward the lipid bilayer to form an ion channel or a pore [160]. A third domain dubbed LLP-3, located between the LLP-1 and -2 domains shows a propensity to form an amphipathic α-helix, but lacks the charged residues on one face, instead possessing a leucine zipper-like sequence [152]. LLP-3 could, in the context of the full protein, span the lipid bilayer to help form the channel. It is also possible that LLP-3 could interact with LLP-1 and 2 in a manner that aids in their aggregation, an assertion the Kliger et al., 1997 based solely on analogy to other leucine zipper-containing proteins.

Studies utilizing synthetic peptides of identical amino acid sequence to the LLP-1 domain have supported the hypothesis that LLP-1 can oligomerize, insert into membranes and form pores. LLP-1 peptides of HIV-1 and SIV were bactericidal to both gram(-) and gram(+) bacteria at micromolar concentrations within a few minutes [151,161-163]. These peptides were also capable of lysing red blood cells (RBC's) and RH9 T-lymphoblastoid cells in similar concentration ranges. LLP-1 peptides were more effective than the natural amphibian cytolytic magainin peptides in these assays. Mutation of 2 – 3 of 7 positively charged arginine residues present in LLP-1 to neutral glutamate residues resulted in an almost complete loss of activity against both prokaryotic and eukaryotic cells in lysis assays [151,161]. Glutamate was chosen to preserve the overall hydrophobic moment of LLP-1 as well as to preserve its secondary structure. Thus, the overall positive charge provided by its arginine residues is most likely necessary for its function.

Experimental evidence supports the theoretical models of the LLP domains' secondary structure and function. Circular dichroism studies show that synthetic peptides corresponding to these regions have little secondary structure in water, but adopt an α-helical secondary structure in the presence of a lipid environment [152,162,164-166]. Transmission electron microscopy studies confirm that, similar to data gathered using magainin-2, LLP-1 interacts with both the inner and outer leaflets of the cytoplasmic membrane of the bacteria *Serratia marcescens *[167]. Bacteria exposed to LLP-1 displayed a decreased cytoplasmic density from negative controls indicating that the membrane had been compromised. Furthermore, addition of membrane impermeable ONPG to LLP-1-incubated, but not control cultures, led to its hydrolysis over time, indicating that it was able to gain access to the β-galactosidase enzyme located in the cytoplasm of bacteria [162]. Both LLP-1 and LLP-2 were able to cause time- and dosage-dependent release carboxyfluorescein entrapped egg PC vesicles at micromolar concentrations. When added to LUV's of various lipid compositions, 15-mer peptides spanning all three LLP regions were capable of causing leakage, phospholipid mixing, and fusion to differing extents [168]. The presence and amount of sphengomyelin as well as cholesterol correlated positively with these peptides' functions in these assays, though similar trends were observed between strains of HIV.

In a subsequent attempt to define the size of the pore created by LLP-1, Miller et al., 1993 measured the amounts of ^45^Ca, ^14^C-sucrose, and ^14^C-inulin that were able to enter LLP-1 treated CEM cell cultures [169]. ^45^Ca (M.W. = 45 Da) and ^14^C-sucrose (M.W. = 342.3 Da), but not ^14^C-inulin (M.W. ~5000 Da) were able to pass through LLP-1 treated membranes, suggesting that LLP-1 could form a pore of a definable size and did not simply destabilize or disintegrate the membrane. In good agreement, membrane perturbation studies utilizing whole virus show that hygromycin b (MW 527) was able to enter cells after infection with HIV-1, while G418 (MW 693) was not able to enter [170]. This suggests that the pore created by the LLP domains has a cutoff between MW 527 and 693.

Topological analysis of full length TM of HIV-1 using sequence specific antibodies showed that not only did TM contain an N-terminal transmembrane anchoring domain, but it also formed secondary associations at the C-terminal end which blocked antibody binding [171]. Furthermore, the association of the cytoplasmic tail with microsomal membranes made from canine pancreas conferred resistance to extraction via carbonate treatment, suggesting that the association observed earlier was not merely an artifact of having a conformational dependent antibody. The association of the cytoplasmic tail with lipid membranes was also resistant to high salt extraction and proteolysis [172]. Expression of just the cytoplasmic tail of TM associated with lipid membranes and was sufficient to get cell surface expression of the tail fragment [173]. Sucrose gradient centrifugation, chemical cross-linking, and gel filtration analysis of an MBP-cytoplasmic tail fusion protein proved the formation of a higher ordered, multimerized structure, dominantly a hexamer. Analysis of the same fusion protein in a mammalian 2-hybrid assay and in a GST pull-down assay complemented these studies in eukaryotic cells [174]. In fact, the authors concluded that the cytoplasmic tail itself is sufficient to oliogomerize Env.

LLP-1 causes increased the conductance of various membranes when added exogenously. LLP-1 bound preferentially to planar lipid bilayers composed of negatively charged phosphatidylserine (PS) over neutral diphytanoyl phosphatidylcholine (DPC) bilayers and as such, all experiments were performed using PS bilayers [175]. At micromolar concentrations there was an overall increased conductance at negative and positive voltages. A preference for cations over anions was observed, with no preference of Na^+ ^or K^+^. The effect of exogenous LLP-1 peptides on whole-cell conductance of (Sf9) insect cells was measured in the same study. Nanomolar LLP-1 concentrations increased mean membrane conductance at positive and negative voltages by approximately 10 fold using the patch clamp technique. Nanomolar concentrations of exogenous LLP-1 were also able to increase the whole cell conductance of *Xenopus laevis *oocytes [176]. Much Smaller conductances were induced by equal concentrations of the lytic peptide melittin. Up to four times the concentration of HIV-1 Nef accessory protein did not increase oocyte membrane conductance over control, untreated oocytes.

Ultrastructural analysis of eukaryotic cells incubated with LLP-1 peptides uncovered features of necrosis as the main cause of death of these cells [177]. The most striking features visible under electron microscopy are extensive vacuolization of the endoplasmic reticulum and mitochondria. The authors attribute this to a concentration of ions and water into these cell organelles attempting to compensate for increased intracellular levels of ions and water caused by LLP-1 peptides. Plymale et al., 1996 also observed a small increase in the levels of apoptosis in these cells [178]. The increase in the numbers of cells undergoing apoptosis under these conditions was very small compared to the increase in cells undergoing necrosis. The magnitude of the increases was influenced by the cell type and the concentration of LLP-1. Lower "sub-lytic" concentrations of LLP-1 tended to cause more apoptosis than higher "lytic" LLP-1 concentrations. Thus apoptosis, like syncytia formation, appears to be a mechanism which HIV can utilize to cause cytopathology, but is not likely the dominant mechanism utilized *in vivo*.

**Figure 6:**
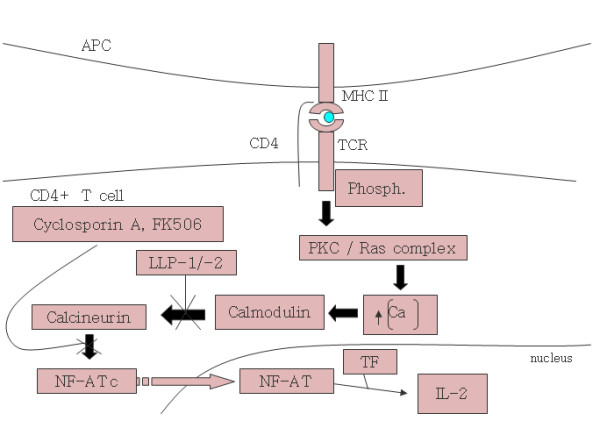
**Calmodulin binding activity of the LLP domains**. Proposed action of LLP-1 and -2 binding of calmodulin in T cell anergy. The LLP domains are thought to disrupt the signaling cascade through titration of calmodulin. Figure was produced based on data from Beary et. al, 1998 [181].

By virtue of its amphipathic α-helical secondary structure, LLP-1 has homology to calmodulin binding proteins and has been proposed as a mechanism behind HIV's ability to cause apoptosis in cell culture. HIV virions which possess full-length TM cytoplasmic tails, but not virions with truncated tails lacking LLP domains, are able to bind calmodulin [179]. Peptides corresponding to both the LLP-1 and -2 domains bind calmodulin with high affinity, irrespective of natural sequence variation present in different clades of HIV [161,180]. When added exogenously to T cells *in vitro*, both LLP-1 and -2 inhibited T cell signal transduction through the NF-AT complex via sequestration and titration of available calmodulin (Figure 6). LLP-1 and -2 were as effective at inhibiting calmodulin as the known calmodulin inhibitor, W-7 [181]. The end result of calmodulin inhibition observed in these studies was a decrease in IL-2 production leading to T cell anergy and increased levels of apoptosis. It has long been observed that immune cells from HIV infected patients are less responsive than HIV seronegative persons though it remains to be seen whether significantly increased HIV-induced apoptosis occurs *in vivo *at all [182]. However, cell culture experiments tend to suggest that apoptosis is not a major contributor to cell death as only marginal increases in apoptotic cells are observed throughout the course of HIV infected cell cultures compared to necrotic cell death [177].

TM has been implicated in altering [ion]_i _thought to be responsible for the impairment of brain function known as AIDS Dementia Complex (ADC). Upon necropsy, neurohistopathology of the CNS shows morphological abnormalities and death of neurons, astrogliosis, microglial nodules, and multi-nucleated giant cells consisting of monocyte-macrophages and microglial cells [55]. Patients with ADC have increased levels of glutamate^+ ^in their CSF [183]. Glutamate^+ ^is an excitatory amino acid (EAA) whose levels are closely regulated by glial cells in the brain because excess glutamate^+ ^in neuronal synapses is toxic. Though neuronal infection is generally non-productive *in vitro*, an increase in extracellular [EAA] is observed after acute infection of neurons by HIV. Analysis of virion proteins proved that TM was sufficient to cause the increased [EAA]_e_. Addition of exogenous peptides with sequences corresponding to the LLP-1 domain of TM had the same effect as the expressed TM protein. It was postulated by Kart et al., 1998 that LLP-1 had its action through ablation of the Na^+ ^gradient, a postulated action of the LLP domains (discussed more in depth below)[183]. Without the Na^+ ^gradient to drive the Na^+^-glutamate^+ ^cotransporter-mediated electrogenic uptake of glutamate^+ ^against its large concentration gradient across the plasma membrane, [EAA]_e _and [cation]_e _increase. Bubien et al., 1995 report that SU added exogenously to cultures of rat or human astrocytes stimulates the Na^+^/H^+ ^exchanger to alkalinize the cytoplasm [184]. This in turn inhibits the Na^+^-dependent uptake of glutamate^+ ^against its concentration gradient and activates pH-sensitive K^+ ^channels to release intracellular K^+^. It is thought that impairment of the astrocytes' ability to maintain the proper [EAA]_e _and [ion]_e _leads to improper firing of neuron potentials and neuronal cell death. It is unknown at this time what the relative contributions of each of these pathways may be *in vivo *to developing ADC.

Previous work on TM and its ability to perturb membranes focused on truncations in the context of whole virions [185-189]. These studies produced conflicting reports of the function and necessity of the C-terminus of TM during infection. Results gained from these truncations vary between producing no effect at all to one or more of decreases in viral entry, infectivity, cytopathic effect, and envelope production, processing, stability, cell surface expression, and virion incorporation. Two points that these studies generally agree on is that HIV virions with truncated TM cytoplasmic tails are replication competent and that most effects observed in these mutant viruses are cell type dependent. Discrepancies between studies likely involve the disruption of multiple domains contained within the cytoplasmic tail of TM, depending upon the extent of each truncation. Several studies have indicated that there may be domain(s) in the C-terminus of TM that interact with HIV Gag proteins to assemble virions [24,190]. In addition to interacting with Gag, it has been hypothesized, though not proven, that the cytoplasmic tail of TM may interact with cellular factors, and that this may be the source of the cell-type dependent effect [191-193].

Studies involving site-directed mutagenesis of specific domains within the cytoplasmic tail may help to clear up some of the confusion caused by the truncation studies. Kalia et al., 2003 engineered infectious virions with mutations of the LLP-1 and -2 domains [194]. Three mutant viruses were produced, one with 2 arginine to glutamate mutations in LLP-1, another with 2 arginine to glutamate mutations in LLP-2, and a third incorporating the same mutations in LLP-1 and -2, but in the same virus. Synthetic peptides corresponding to these domains which include these mutations were previously found to lose their lytic properties, as well as their calmodulin binding activity [169,180]. The LLP-1 mutant virions displayed an approximate 85% decrease in TM incorporation, though Env expression and processing were unaffected. LLP-2 mutant viruses were unaffected in Env expression, processing, oligomerization, and incorporation. Both LLP-1 and -2 mutant viruses were decreased in their capacity to cause syncytia by 70 and 90% respectively. Double mutant viruses were similar to LLP-1 mutant viruses in envelope expression, processing, and incorporation, but failed to cause syncytia to the same extent as LLP-2 mutant viruses [194].

Syncytia formation and single cell balloon degeneration have traditionally been thought to be two distinct cytopathic phenotypes caused by two distinct regions of Env – the extracellular fusion peptide and the intracellular LLP domains respectively. However, these two processes may be more closely linked than previously thought. Giant, multinucleated syncytial cells undergo increases in total cell volume, owing to the LLP domains perturbing the plasma membrane just as in the case of single cells. On the other hand, Kalia et al., 2003 report that infectious clones containing 2 each of arginine to glutamate site directed mutations in the LLP-1 and/or LLP-2 domains exhibit a decrease in their ability to cause cell:cell fusion [194]. It is possible, though it remains to be proven, that the membrane perturbation properties of the LLP domains could synergize with the fusion peptide to increase the efficiency of cell:cell fusion in a manner analogous to its function in virion budding. Increases in cell volume due to osmotic balancing after ion influx could disrupt the cell cytoskeleton allowing for greater ease of membrane fusion.

The case is more clear-cut for truncations of the cytoplasmic tail of the Env protein of SIV. Env truncations are documented to arise during culturing of SIV in human cell lines [195]. Mutants revert back if cultured again in simian cells [195,196]. While these SIV TM truncation mutants are replication competent *in vitro *as well as *in vivo*, they lack full pathogenicity *in vivo *and tend to revert back over time [197]. Shacklett et al., 2000 showed that the cytoplasmic tail of SIV Env is necessary for persistence of viremia and pathogenesis of the virus in rhesus macaques [198]. Their group engineered 3 stop codons, a +1 frameshift mutation, and 3 arginine to glutamate site directed mutations in the LLP-1 domain of mac239 virions, eliminating both LLP-1 and -2 regions. The resulting mutant virions cause an initial viremia, but levels fall off and become non-detectable over time. All viruses recovered from these macaques maintained their mutations without exception and none of the infected macaques developed SAIDS. Juvenile macaques infected i.v. with this mutant virus maintained low level viremia, but also never progressed to SAIDS. 100% of macaques infected with wild type mac239 develop SAIDS over the same time course.

### LLP viroporin models and discussion

If the LLP domains form a viroporin and allow ions to enter the cell more freely down its concentration gradient, then water would follow in attempt to osmotically balance those ion influxes. This would represent a mechanism by which HIV-1-infected cells undergo the process of balloon degeneration.

This hypothesis allows for a mechanism by which large versus moderate ion influxes could direct differential outcomes for infected cells [199]. Those cells that can overcome the osmotic imbalance may then live to become chronically infected. Those that can't overcome this osmotic imbalance undergo balloon degeneration. In support of this theory, the concentration of LLP peptides exogenously added to mammalian cell culture were previously determined to correlate with a differential outcome on cell death [178]. The levels of apoptosis versus necrosis of these cells – higher concentrations (>100 nM) were shown to result in more necrosis, while lower concentrations (~20 nM) resulted in more apoptosis with exogenous LLP-1.

It is unknown to what extent single cell killing versus syncytial cell death occurs *in vivo*. The central nervous system is the only tissue where syncytial cells have been observed *in vivo *at autopsy [55,200]. In the absence of observed syncytia, it is assumed that single cell death occurs in the rest of the body, possibly due to a lack of opportunity for infected cells to be in close enough proximity to allow syncytial formation. As an estimate for the amount of single cell death that can be caused by Env in the absence of syncytia formation, 43% of RH9 T-lymphoblastoid cells died by single cell death after inducibly expressing Env in the presence of soluble CD4 to prevent syncytia formation [199].

There are three general mechanisms by which the LLP peptides and expressed Env may act to alter oocyte membrane permeability. They may be acting to modify endogenous ion channel function, altering its activity and thereby increasing whole-cell conductance. This could occur through direct contact or by shifting the intracellular ionic environment. For example, LLP-1 is known to have the ability to bind calmodulin leading to an increase in intracellular free calcium [161,179,180]. Calcium levels have a large effect on protein activation in the cell, thus the LLP domains could be working through this mechanism exclusively, or in addition to forming a membrane pore. Second, the LLP domains could act to nonspecifically disrupt the membrane, or specific regions of the membrane in a detergent-like effect. Lastly, the LLP domains may serve as viroporins i.e., insert into the membrane to form pores. Mounting evidence suggests that the last possibility is likely; however definitive differentiation of these mechanisms would require the discovery of specific inhibitors, such as amantadine for the M2 channel of influenza.

When partitioning into a lipid environment, LLP-1, -2, and -3 form highly ordered α-helices that were able to disrupt a variety of model lipid bilayers in the absence of all other protein (discussed above). Consequently these domains are capable of disrupting membranes and are not solely dependent upon alteration of endogenous ion channel function to alter the intracellular ionic environment. In the context of a living membrane, LLP-1 and -2 have been shown to lyse bacteria, fungus, red blood cells, and various cultured eukaryotic cells [151,162,169,178-180,201,202]. When added exogenously, LLP-1 can increase the conductance of *Xenopus *oocytes, presumably caused by the formation of transmembrane pores which increase the membrane permeability of electrogenically active ions [176]. It has thus been postulated that LLP-1, and possibly LLP-2 peptides, oligomerize to form a "barrel-stave"-like pore, which are conducting pores (barrels) in membranes formed by the self-assembly of a variable number of alpha-helical rods (staves). It is the formation of these pores which then allow ions to be redistributed across the membrane to cause osmolysis.

Recently it has been proposed that HIV, along with several animal viruses including influenza, may utilize lipid rafts to enter and exit cells. Lipid rafts are lipid microdomains enriched for sphingomyelin and cholesterol that are thought to function by aggregating proteins that require close proximity for proper function, such as in the case of the T cell synapse [203]. It has been proposed by several groups that HIV-1 exploits lipid rafts during both the entry and budding stages of its replication cycle. During entry, lipid rafts may serve to concentrate cellular receptors (CD4, CXCR4, and CCR5) for ease of entry by HIV [204]. This could potentially be important for HIV since entry has been shown to be dependent on the density of receptors on the cell surface [205].

There is ample evidence that HIV-1 buds from lipid rafts [28,206]. The composition of the HIV envelope is primarily sphingolipid and cholesterol, resembling the composition of rafts. The HIV lipid envelope is furthermore enriched for proteins known to partition into lipid rafts and excludes proteins known to not associate with rafts (such as CD45). HIV proteins extract with lipid raft domains during nonionic detergent extraction at 4°C. Lastly, the cytoplasmic tail of Env has two palmitoylated sites known for targeting proteins to lipid rafts in a manner analogous to cellular proteins' targeting to lipid rafts [207]. One result of entry and budding through lipid rafts is that Env proteins would be concentrated in distinct areas on the cell surface, possibly allowing interaction of LLP domains to form pores on the cell surface. These pores in turn could weaken the normally stable raft area through altered intracellular ion milieu, thereby creating a more favorable environment for replication and budding of the virus. Hence, as infection progresses and increasing amounts of Env are deposited on the cell surface in preparation for budding, there would be a concomitant increase in cytopathic effects, such as intracellular ion imbalance, cell volume dysregulation, and balloon degeneration.

Prior observations that LLP-1 can bind to intracellular signaling molecules, such as calmodulin to ultimately induce apoptosis and/or necrosis [161,178-180] suggest that the LLP domains may be configured in certain situations as a pore passing through the membrane and part of their time associated with the inner leaflet of the lipid membrane where they are able to interact with these intracellular molecules. Flip-flopping between lipid bilayers of amphipathic pore forming peptides has been documented with melittin [208,209]. Based on reported similarities between melittin and LLP peptides, it is reasonable to hypothesize that the LLP domains may be flip-flopping between a transmembrane state and parallel association with the inner leaflet of the lipid bilayer. On the other hand, the LLP domains may possess different activities in the different cell types that it infects, or there may be some as of yet undefined temporal control that allows these two alternate functions to take place at appropriate times during infection.

Since the LLP domains are also present in the context of the virion, it is possible that they would have an effect at this stage of the HIV replication cycle. There is at least one report of an increase in natural endogenous reverse transcription (NERT) cause by the LLP domains increasing the virion envelope permeability to dNTP's [210].

That HIV may code a viroporin in its major surface glycoprotein would ensure that the membrane perturbation, ion fluxes, volume changes, and resulting "loosening" of the plasma membrane and cytoskeleton always occur when and where it is needed for budding, syncytial formation, and/or single cell balloon degeneration. Concentrating HIV glycoproteins in lipid rafts could allow for localized unstable membrane regions at the exact points where it is needed by HIV. While it seems possible that Vpu could also act at these stages to accomplish the same goals, it is more difficult to envision how it could accomplish the task as Vpu has been shown to be excluded from the plasma membrane and HIV virions [75,99].

Since SIV does not contain an equivalent of Vpu in its genome (except SIV_cpz_), there is some conjecture in the literature that it utilizes its Env protein and specifically the LLP domains of Env to take the place of Vpu in enhancement of virion budding [211]. In this situation it makes sense that deleting the LLP domains in SIV makes a more clear cut phenotype *in vivo *than HIV-1. SIV minus the LLP regions is still able to replicate, but does not cause SAIDS [198]. It may be that the LLP domains of SIV are functional equivalents of Vpu in the case of SIV. By extension, it is possible that these two proteins represent backup systems in HIV-1 for budding, or maybe that Vpu and LLP have specialized such that Vpu helps Env traffic through the ER and Golgi while the LLP's increase budding more specifically through cytoskeletal disruptions at the cell surface. Though admittedly it is not understood to what extent deletion of LLP domains in HIV-1 would do *in vivo *since that type of controlled experiment cannot be done ethically – truncated mutants have been found in productive human infections which cause AIDS and make it difficult to dissect the roles of these two proteins in infection [212,213].

In addition to the LLP's involvement as a backup system for cell volume regulation and cytoskeletal disruption, they may produce secondary effects, such as AIDS-related dementia complex and bystander cell death. LLP domains could be cleaved by cellular proteases from the C-termini of TM proteins and act as exogenous peptides for all intents and purposes *in vivo*. In this way they could produce the effects generated by LLP in cell culture thought to cause AIDS-related dementia (described above). An analogous role could be played in the death of bystander cells – a population of cells that die in HIV-infected individuals, but are not productively infected [214,215].

The Lentiviral Lytic Peptide motif may not be unique to lentiviruses, as their name would imply. Recently it was discovered that the carboxyl termini of pestivirus envelope glycoproteins contain a sequence predicted to have a high propensity to form an amphipathic α-helix [216]. This sequence is conserved among pestiviruses. It remains to be proven that this region of pestiviruses employ any functional characteristics of LLP's, however it could be hypothesized that a functional motif is beginning to show itself. Functional substitutions of 6K protein of sindbis virus and Vpu of HIV-1 have been proven [116]. Thus it begs the question, could the 6K protein and LLP domains of the E1 surface glycoprotein of sindbis virus be functional equivalents of the Vpu protein and the LLP domains of the Env surface glycoprotein of HIV-1? Functional equivalents may exist in other viruses, such as the P7 protein and LLP domains of BVDV (also a pestivirus), as well as the VP1 and 2B proteins of poliovirus, a picornavirus [64,217,218].

In 2004 alone it was estimated that there were approximately 39.4 million people living with HIV/AIDS, with around 3.1 million AIDS related deaths, and 13,500 new infections each day [222]. Even with the advent of Highly Active Anti-Retroviral Therapy (HAART), which combines the use of protease inhibitors and reverse transcriptase inhibitors, and use of the newer fusion inhibitors such as T20, HIV continues to be a serious threat to world health [219,220]. A lack of resources for most infected persons to purchase the drugs, the intensive treatment regimen, the toxicity of drug regiments, and emerging drug resistance all contribute to a lack of general efficacy of the current treatment regimen and highlight the necessity for more basic research with the ultimate goal of development of new treatments. The LLP domains may represent a new target for HIV drugs to inhibit HIV infection. Otherwise the development of eLLP's as a new class of antibacterial drugs could be used to help resolve AIDS-related infections, as well as serve as a new class of antibiotics – virally derived antibiotic peptides.

In order to develop the LLP domains as an attractive target for the development of novel anti-HIV therapies, it will likely be necessary to achieve a better understanding of the mechanism of action of this domain. The use of biochemical techniques such as oriented circular dichroism (OCD) spectroscopy could be used to determine the orientation of LLP peptides in lipid membranes, either spanning the membrane, lying on the surface, or somewhere in between. Expanding on the experiments of Comardelle et al., 1997 and continuing to define the effects of LLP peptides on *Xenopus *oocyte membranes could also be helpful as this system was helpful in defining the activity of the M2 protein of influenza. Ion-selective electrodes could be employed to characterize the ion selectivity of LLP peptide domains. Specific LLP domain mutations could be made to dissect each LLP domains' role in the proposed viroporin. Specific inhibitors of the LLP domains could be screened for, which could help prove specific activity of the LLP domains as a viroporin and could themselves be a candidate for a novel class of anti-HIV drugs.

## Summary of Results

Mechanisms by which HIV-1 mediates reductions in CD4^+ ^cell levels in infected persons are intensely investigated, and have broad implications for AIDS drug and vaccine development. Virally induced changes isn membrane ionic permeability contribute to cytopathogenesis induced by lytic viruses of many families. HIV-1 induces disturbances in plasma membrane ion transport. The carboxyl terminus of TM contains amphipathic α-helical motifs identified because of their structural similarities to melittin, a naturally occurring cytolytic peptide, and were dubbed lentiviral lytic peptides (LLP) -1, -2, and -3. Peptides corresponding to these domains from HIV-1_HXB2 _(a clade B laboratory adapted virus) partition into lipid membranes as α-helices and disrupt model lipid membranes. A peptide corresponding to the LLP-1 domain of a clade D HIV-1 virus, dubbed LLP-1D displayed similar activity to the LLP-1 domain of the clade B virus in all assays, despite a lack of amino acid sequence identity. When individual peptides are incubated exogenously with *Xenopus *oocytes, LLP-1 and -2, but not LLP-3 increased the whole cell conductance across the plasma membrane. The increased conductance observed with LLP-1 and -2 appears to be at least in part due to an increased permeability of Na^+ ^ions. A peptide corresponding to the LLP-1 domain of a clade D HIV-1 virus, dubbed LLP-1D, displayed similar activity to the LLP-1 domain of the clade B virus in all assays, despite a lack of amino acid sequence identity.

Combinations of LLP peptides appear to act cooperatively to increase the whole cell conductance of *Xenopus *oocyte plasma membranes. Taken together, these results suggest that the C-terminal domains of HIV-1 *Env *proteins may form an ion channel, or viroporin, that is capable of conducting Na^+ ^ions. Alternatively, HIV-1 *Env *protein may activate a silent *Xenopus *oocyte Na^+^-conductive ion channel. Increased understanding of the function of LLP domains and their role in the viral replication cycle could allow for the development of novel HIV drugs.

## Biography

Joshua Costin was born in Colorado Springs, CO on July 14, 1976. He grew up in the tropical paradise that is Naples, Florida and graduated 7th in his class at Naples High School. He went on to graduate magna cum laude from Florida State University, with honors, as well as honors in the major, receiving a B.S. in Biology, a B.S. in psychology, and a minor in chemistry. Joshua enrolled in the Department of Microbiology and Immunology at Tulane University in the fall semester of 1998 and began what was to become his dissertation work in November of that year under the guidance of Dr. Robert F. Garry and completed his dissertation work in the summer of 2005. Along the way Joshua received several awards, including a Tulane University Cancer Center Grant in 1999, the Center for Infectious Diseases Award for Basic Research in Infectious Diseases 2001, and a Graduate School Dissertation Year Fellowship for Sciences and Engineering for 2004–2005.

## Competing interests

The author(s) declare that they have no competing interests.

## Authors' contributions

JC researched and wrote the review article.
